# Deep learning–based non-invasive prediction of axillary lymph node metastasis in breast cancer: performance of the YOLO-v11 object detection algorithm

**DOI:** 10.1186/s12880-026-02289-0

**Published:** 2026-03-16

**Authors:** Sevgi Ünal, Remzi Gürfidan, Merve Gürsoy Bulut, Oğuzhan Kilim

**Affiliations:** 1 Department of Radiology, İzmir Katip Celebi University Ataturk Training and Research Hospital, Izmir, Türkiye; 2 Isparta University of Applied Science, Isparta Vocational School of Information Technologies, Database, Network Design and Management, Isparta, Türkiye

**Keywords:** Breast cancer, Axillary lymph node metastasis, Ultrasound YOLO-v11 Models, Machine learning

## Abstract

**Background:**

This study proposes a deep learning-based decision support system for the non-invasive prediction of axillary lymph node (ALN) metastasis in breast cancer using ultrasound (USG). Both localisation and benign–malignant classification of ALNs were performed using YOLO-v11, a single-stage object detection approach. The dataset created from labelled USG images was split into training/validation/test sets at a ratio of 70%/20%/10%, and the model produced a class label and confidence score for each prediction. Performance was found to be high with amAP@0.5 = 0.904s (benign: 0.866, malignant: 0.942); F1-, precision-, and sensitivity-confidence curves showed that thresholds in the 0.52–0.60 range provided a balance between sensitivity and specificity. Confidence scores for the malignant class were observed to be concentrated in the 0.82–0.87 band. The confusion matrix includes an auxiliary ‘background’ row/column representing unmatched predictions (false positives) and missed ground-truth detections (false negatives); background was not modelled as a semantic class during training. The findings point to a rapid and reproducible artificial intelligence approach that could reduce operator dependency in USG. The proposed model has the potential to provide radiologists and clinicians with non-invasive, real-time decision support in ALN assessment.

**Objective:**

Axillary lymph node (ALN) metastasis plays a decisive role in the clinical course and treatment planning of breast cancer following diagnosis. Therefore, the accurate and early detection of metastatic lymph nodes directly impacts treatment success. Ultrasonography (USG) stands out as the most common and effective imaging method for evaluating axillary lymph node metastasis. The main objective of this study is to develop an artificial intelligence-supported USG analysis system to provide a decision support system for radiologists and clinicians in the detection of ALN metastasis.

**Methods:**

This study included a total of 471 patients: 248 patients diagnosed with breast cancer between 2024 and 2025 and reported as malignant based on axillary lymph node biopsy results, and 223 control cases with benign lymph nodes detected in breast ultrasound scans. Using a dataset created from the acquired ultrasound images, a single-stage object detection model (YOLO-v11) was employed to detect ALN metastasis. The data were split into a 70% training, 20% validation, and 10% testing ratio. The developed model produces a confidence score and class label for each detection.

**Results:**

The developed model has demonstrated high accuracy performance in benign and malignant lymph node samples. The mAP@0.5 value calculated under the Precision–Recall (PR) curve was obtained as 0.904 (benign: 0.866, malignant: 0.942). The precision and sensitivity values were found to be 0.866 in benign samples and 0.942 in malignant samples, respectively. Confidence score values for the malignant class were concentrated in the range of 0.82–0.87. When evaluated from a clinical application perspective, the threshold value in the range of 0.5–0.6 provides a balanced trade-off between sensitivity and specificity.

**Conclusion:**

The proposed artificial intelligence model can localise axillary lymph nodes with high accuracy on USG images and distinguish between benign and malignant conditions. In this respect, the model has the potential to guide radiologists and clinicians as a decision support tool in the diagnostic process.

## Introduction

According to the GLOBOCAN 2020 cancer incidence and mortality estimates published by the International Agency for Research on Cancer (IARC), breast cancer in women is reported as the most commonly diagnosed malignancy worldwide, with approximately 2.3 million new cases (11.7%) [1]. Breast cancer has a high metastatic potential in approximately 20–30% of cases, most commonly spreading to the axillary lymph nodes (ALNs) [2]. Axillary lymph node metastasis is one of the factors that determine the pathological stage of breast cancer and has the strongest impact on prognosis and survival [3]. Furthermore, preoperative axillary assessment is critical in determining systemic treatment options, planning the surgical approach, and deciding on postoperative radiotherapy [4]. Therefore, accurate assessment of ALN involvement is a fundamental component of staging and appropriate treatment planning in breast cancer patients [5].

In early-stage breast cancer, axillary surgical assessment is performed using sentinel lymph node biopsy (SLNB), a less invasive and more protective method [6, 7]. The American College of Surgeons Oncology Group (ACOSOG) Z0011 study demonstrated that SLNB alone is sufficient in patients who are clinically node-negative, have undergone breast-conserving surgery, and have one or two nodal metastases [7]. Guidelines recently published by the National Comprehensive Cancer Network (NCCN) state that axillary assessment can be performed using USG or other imaging methods [8]. Furthermore, it is recommended that axillary USG or breast MRI can be used in patients who will undergo neoadjuvant chemotherapy (NAK) and in cases where pathological complete response (pCR) assessment is required after treatment [8]. Cases with low tumour burden have been considered candidates for SLNB in the surgical axillary staging algorithm [8].

USG is the primary diagnostic method for evaluating axillary lymphadenopathy and also allows for interventional procedures such as fine needle aspiration biopsy or tru-cut biopsy [9]. Various ultrasonographic criteria, such as size, shape, cortical thickening, and loss of fatty hilum, are used to characterise malignant lymph nodes [10]. However, as USG is a user-dependent method, operator dependency constitutes a significant limitation in the evaluation of ALN. Furthermore, no suspicious ultrasonographic features can be detected in approximately 35% of metastatic ALNs [11].

In recent years, numerous studies have been conducted on machine learning-based approaches in the field of medical imaging. The most commonly used methods in these studies have been radiomic analysis and convolutional neural networks. Radiomic analysis is based on the principle of obtaining numerous manually extracted imaging features and using them in machine learning-based classification processes [12]. There are studies demonstrating the potential value of quantitative radiomic features obtained from medical images for predicting ALN status [13, 14].

Studies have shown that deep CNN-based approaches can achieve the most up-to-date performance levels in lesion detection and cancer diagnosis [15]. Among object detection algorithms, Region-based Convolutional Neural Networks (R-CNN), Fast R-CNN, Single Shot MultiBox Detector (SSD), and You Only Look Once (YOLO) models stand out [16]. The YOLO algorithm can perform both localisation and classification tasks simultaneously by enclosing lesions within a bounding box. Compared to two-stage models, the YOLO series offers a more practical, single-stage, and efficient structure in terms of accuracy and speed [17].

According to existing research and literature reviews, there is no study using a YOLO-based model on ultrasound images to predict axillary lymph node metastasis in breast cancer. In this regard, the aim of this study is to develop an artificial intelligence-supported non-invasive diagnostic model based on the YOLO-v11 architecture that could assist radiologists and clinicians in the evaluation of ALN metastasis.

## Materials and methods

### Ethical approval

This study has been approved by the Clinical Research Ethics Committee of Atatürk Training and Research Hospital, İzmir Kâtip Çelebi University. All research procedures involving human participants have been approved by the institutional and/or national research committee and conducted in accordance with the ethical principles of the Declaration of Helsinki. Written informed consent has been obtained from all participants.

### Dataset

The study included a total of 471 patients: 248 patients diagnosed with breast cancer between 2024 and 2025 and reported as malignant based on axillary lymph node (ALN) biopsy results, and 223 control cases with benign ALN detected in breast ultrasound scans.

Each image contained at least one annotated lymph node. In cases where multiple lymph nodes were visible within the same ultrasound frame, each lymph node was annotated separately with an independent bounding box and class label (benign/malignant). The dataset was divided as shown in Table 1.

**Table 1:** Division statistics of the dataset

Subset	Images (*n*)	Annotated LNs (*n*)
Training (70%)	400 (200 benign + 200 malign)	471 (223 benign + 248 malign)
Validation (20%)	129 (68 benign + 61 malign)	150 (76 benign + 74 malign)
Test (10%)	32 (19 benign + 13 malign)	37 (23 benign + 14 malign)

To prevent data leakage and overestimation of model performance, dataset splitting was performed at the **patient level** rather than at the image level. All ultrasound images belonging to a single patient were assigned exclusively to one subset (training, validation, or test).

No images from the same patient were distributed across different subsets. Therefore, the independent test set consisted entirely of unseen patients, ensuring true external validation within the study cohort.

Inclusion criteria were defined as follows:

- Being aged 18 years or older,
- Having a histopathologically confirmed diagnosis of invasive breast cancer in malignant cases,
- Having an ALN biopsy result reported as breast cancer metastasis.

The benign group included cases with no history of malignancy, no suspected pathological lymph nodes in the axillary region, and those evaluated for routine screening purposes.

### Exclusion criteria

Cases with the following characteristics were excluded from the study:

- Patients with a history of chemotherapy or endocrine therapy,
- Pregnant or breastfeeding women,
- Cases with nipple discharge or skin disease,
- Patients with incomplete clinical-pathological data,
- Cases with suspected infection or mastitis,
- Ultrasonographic data with poor image quality or artefacts.

### Ultrasonography examination

All ultrasound (USG) examinations and biopsy procedures were performed by two radiologists experienced in breast radiology. Imaging was performed using two different systems:

- Samsung RS85 Prestige (Samsung Medison, Seoul, Korea).
- Esaote-MyLab 9exp (Esaote S.p.A., Genoa, Italy).

The L12-5 linear probe (12 MHz) was used in all examinations, and the gain, focus and zoom settings were adjusted to obtain optimal image quality for each case. Following 2D ultrasound, artefact-free, high-resolution images were selected for inclusion in the analysis. All breast ultrasound images were extracted from the systems and converted to DICOM format.

Although ultrasound images were acquired using two different systems (Samsung RS85 Prestige and Esaote MyLab 9exp), all data were collected from a single institution. Ultrasound imaging is inherently susceptible to domain shift due to device-specific signal processing pipelines, gain settings, speckle patterns, and operator-dependent acquisition techniques. Because no leave-one-device-out validation or external multicentre testing was performed, the current results reflect internal validation performance rather than full real-world generalizability. Therefore, the robustness of the model across different clinical environments, ultrasound manufacturers, and imaging protocols remains to be confirmed. Future work will focus on multicentre data collection and cross-device validation strategies to evaluate domain adaptation and improve external robustness.

No external filtering or image enhancement was applied prior to model training. All preprocessing operations were handled using the built-in Ultralytics YOLOv11 pipeline to ensure methodological consistency and reproducibility.

### Image labelling and pathological evaluation

The labelling of malignant and benign lymph nodes was performed independently by radiologists with over five years of experience. Pathology reports were evaluated by pathologists experienced in breast pathology.

### Material and method for machine learning

**Fig. 1:**
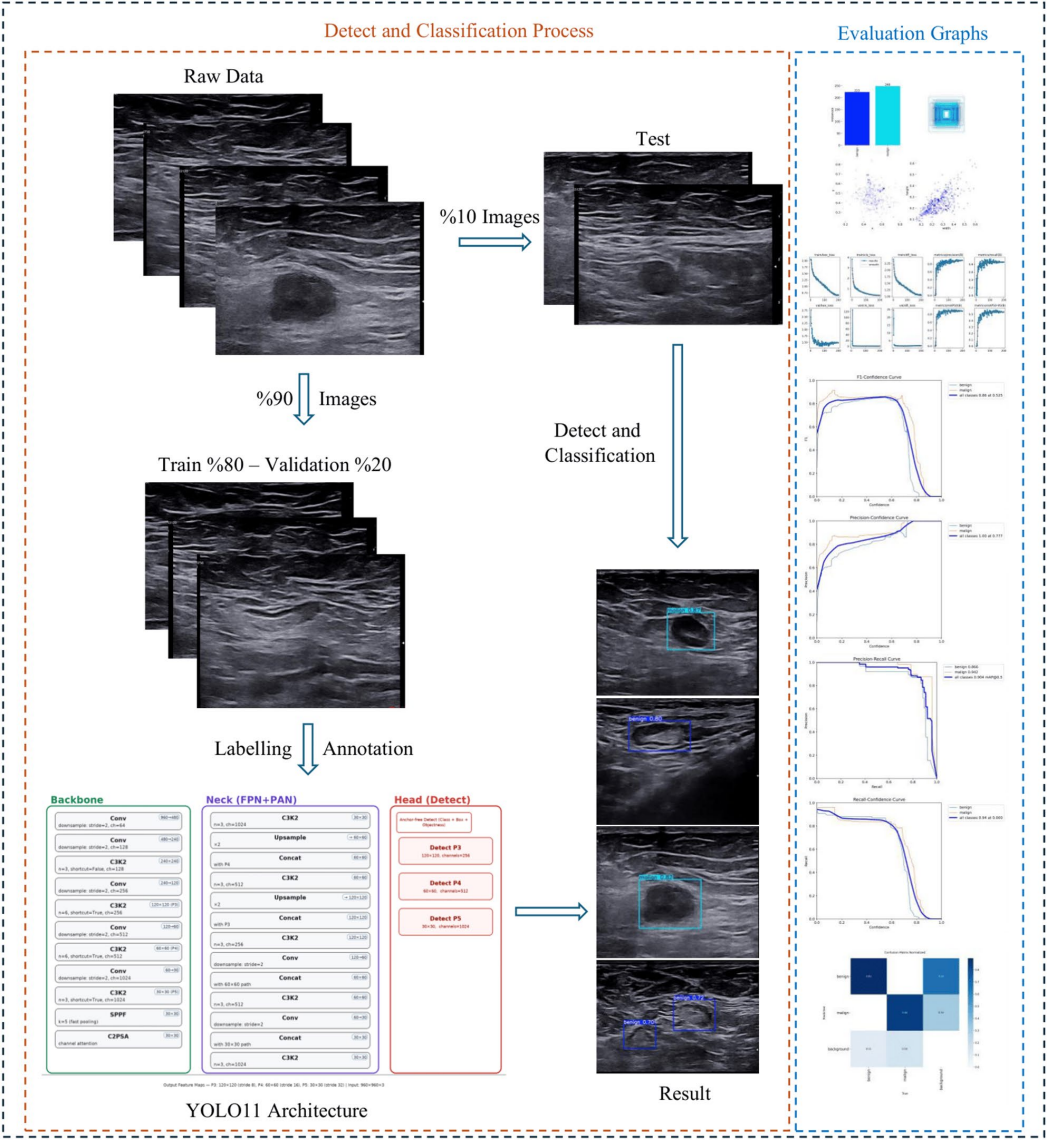
All system architecture

The dataset is annotated with bounding boxes and class labels (benign/malignant) for each image. All system architecture is shown in Fig. 1.

All images were resized to 960 × 960 pixels using YOLO’s letterbox resizing strategy while preserving aspect ratio. Padding was applied where necessary to prevent geometric distortion.

Pixel intensity values were internally normalized by scaling from the original 0–255 range to 0–1. No dataset-specific mean–standard deviation normalization or ImageNet-based normalization was applied.

No additional preprocessing such as contrast enhancement (CLAHE), denoising, sharpening, or histogram equalization was performed. The model was trained directly on clinically acceptable raw ultrasound images.

Data augmentation was implemented using the default Ultralytics YOLO training pipeline. This included geometric transformations and scale-based augmentations. Rectangular training (rect=True) was enabled to minimize artificial padding artefacts during training.

To quantify statistical uncertainty arising from the relatively small independent test set, confidence intervals (95% CI) for primary performance metrics (precision, recall, F1 score, and mAP@0.5) were estimated using bootstrap resampling (1,000 iterations). Resampling was performed at the image level while preserving patient-level independence across subsets.

The data is split into training and validation at 20% and 80%. The model produces a confidence score and a class label for each detection. Performance was evaluated with Precision, Recall, F1, PR curves and mAP@0.5 and mAP@0.5–0.95 metrics. Confusion matrices are reported to see the types of errors. F1-confidence, Precision-confidence and Recall-confidence curves are derived to guide threshold selection. Example result visualisations show typical correct detections and class judgements. The recordings obtained during training show a steady decrease of losses and a plateauing of the validation metrics.

## Results

**Fig. 2:**
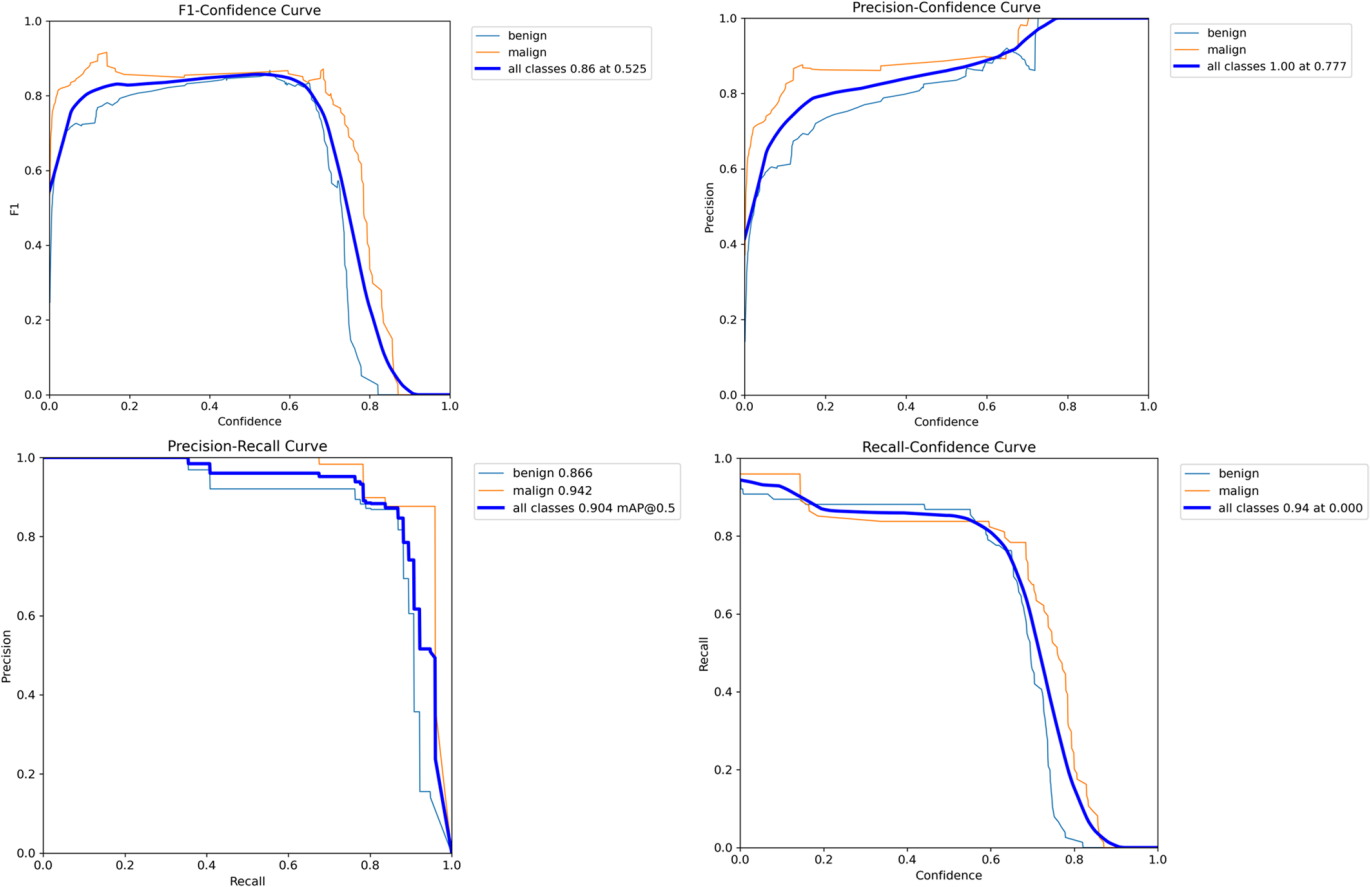
Threshold screening and performance curves by class (benign/malignant)

The four graphs shown in Fig. 2 show from different angles how the model behaves as the “confidence threshold” changes and provide a scientific basis for the choice of threshold.

The F1-Confidence Curve gives the threshold-dependent profile of the F1 score, which is the harmonic mean of precision and recall. Since F1 penalises both false positives and false negatives at the same time, it is used to determine the best “general purpose” threshold. In the graph, F1 for all classes plateaus broadly in the range 0.30–0.65 and peaks at F1 ≈ 0.86 at a threshold of approximately 0.52–0.55. This indicates that there is flexibility in the choice of threshold, but that F1 starts to fall rapidly above 0.70. Complementary to this, the Precision-Confidence Curve shows how precision, which is sensitive to false positives, rises relative to the threshold. As expected, the precision increases monotonically and approaches 1.0 around 0.77–0.78, meaning that this threshold and above produces almost no false positives. In the class breakdown, the malignant curve exceeds the benign curve at most points, suggesting that malignant foci are more visually/texturally distinguishable; they maintain accuracy even at high threshold. The third graph, the Precision-Recall (PR) Curve, presents the trade-off between precision and recall in a single plane by sweeping the threshold and is summarised by the value of mAP@0.5 under the field. It has a numerical value of mAP@0.5 = 0.904 (benign 0.866, malignant 0.942). A prolonged stay in the upper region of the curves indicates that a high accuracy-sensitivity balance can be maintained over a wide threshold range. The higher PR performance of the malignant class is consistent with the previous precision finding. Finally, the Recall-Confidence Curve isolates only the threshold dependence of sensitivity and is particularly critical for screening scenarios. The graph shows that while recall ≳ 0.9 is maintained at low thresholds, the sensitivity decreases rapidly after a threshold of approximately 0.65–0.75. The steeper fall for benign indicates that raising the threshold first increases the risk of benign abduction, whereas in malignant the sensitivity remains slightly more resistant. Interpretation of these four graphs together clarifies the practical threshold recommendation. For general purpose and balanced use, the confidence band of about 0.52–0.60 captures the F1 peak and provides a good balance of precision-recall. In cases with a high false positive cost, going to a threshold of about 0.75–0.80 increases the precision to about 1.0.

**Fig. 3:**
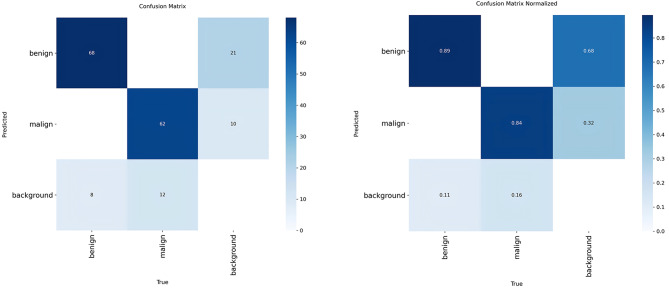
Complexity matrix of classification

Figure 3 shows the raw confusion matrix on the left and its column-normalised counterpart on the right. The columns show the true class, the rows the class predicted by the model; the normalised version scales the columns by 1, allowing us to see the proportion of instances of each true class that go to which class. When read together, these two views clarify both the absolute magnitude and the relative distribution of misclassifications.

**Fig. 4:**
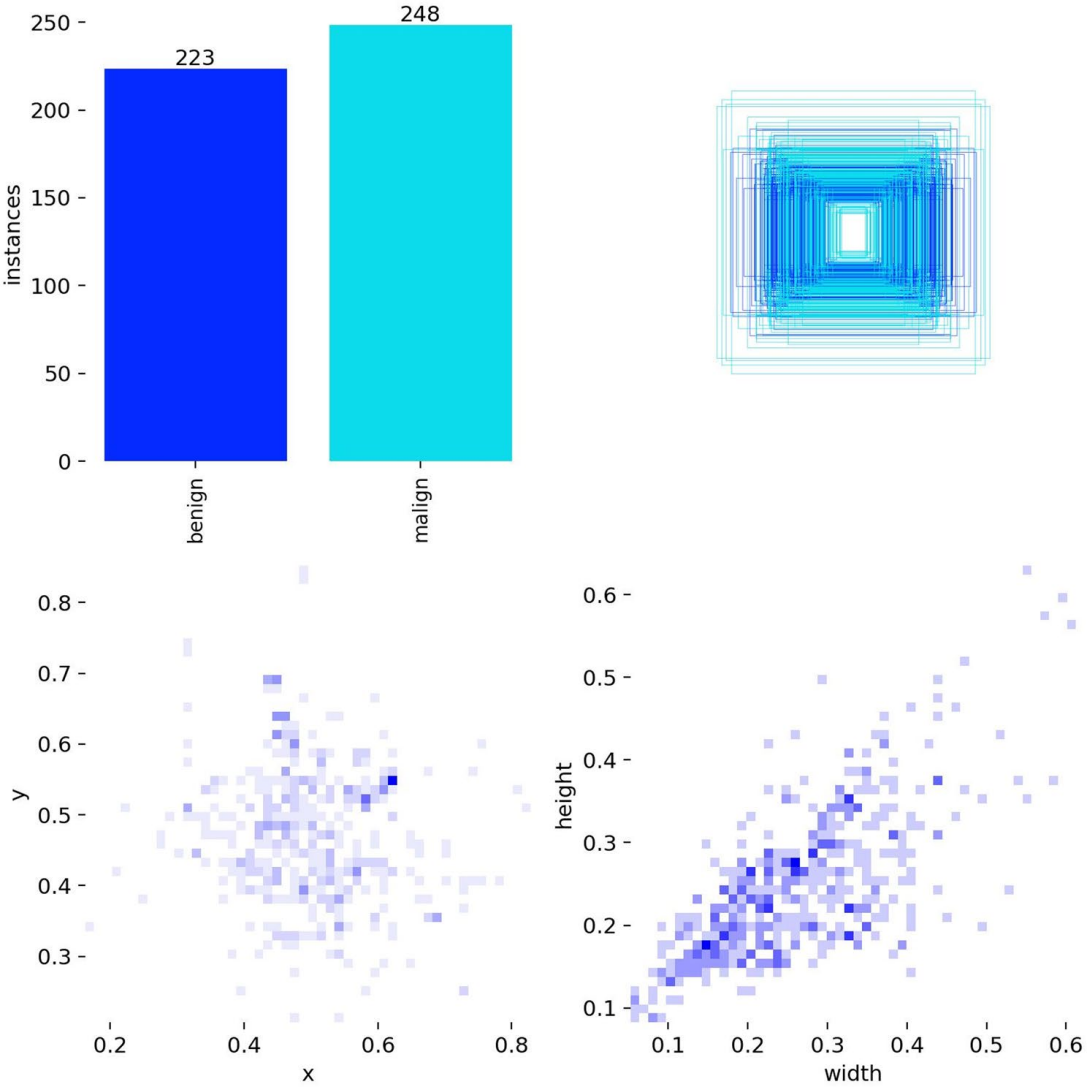
Class, location and size distributions of the lymph node dataset

The model produces generally high accuracy in benign and malignant samples. In the raw matrix, there are 68 correct classifications in the benign→benign cell and 62 correct classifications in the malignant→malignant cell. In the normalised matrix, this corresponds to approximately 0.89 for benign and 0.84 for malignant. The model used in the study was able to discriminate benign cases somewhat more consistently, while the malignant class experienced relatively more deviations. The main problem arises in the confusion with the background. The normalised matrix on the right shows that 68% and 32% correspond to the class distribution of unmatched predictions rather than true background samples. In total, 31 detections did not sufficiently overlap with any annotated lymph node and were therefore considered false positives; among these, 21 were predicted as benign (68%) and 10 as malignant (32%). These values do not indicate that background was learned as an independent class. Instead, they reflect the class labels assigned to detection-matching errors during evaluation. In standard YOLO-based object detection frameworks, background is not defined as a semantic class and is not learned through the classification head. Instead, it is handled implicitly through the objectness score and IoU-based matching mechanisms during training. In the confusion matrix presented in Fig. 3, the “background” row and column do not indicate that background tissue was explicitly classified as benign or malignant. Rather, they represent evaluation artefacts corresponding to predictions that did not sufficiently overlap with any ground-truth annotation (false positives) and annotated lymph nodes that were not detected by the model (false negatives). Therefore, background was not modelled as an independent class, and the benign–malignant distinction was performed exclusively on matched detections. The linear and repetitive texture of ultrasound can also be confused with the oval-hypoechoic node appearance, increasing false positives with loss of context, especially in small boxes. In addition, background-induced False-Positives increase rapidly at low safety threshold when thresholding is not set correctly. Class, location and size distributions of the lymph node dataset is shown in Fig. 4.

The class distribution dataset on the top left is balanced in terms of class balance with ≈ 223 benign and ≈ 248 malignant samples. This balance makes training possible without any special intervention to the class weights. The box-top view in the upper right shows that most of the samples are clustered in the centre of the image and the number of samples becomes sparse towards the edges. The centre distribution x-y heat map in the bottom left shows that the centres are concentrated approximately in the band x∈ [0.35–0.6], y∈ [0.35–0.6]. The size distribution in the bottom right shows that the box width-height points are significantly positively correlated and mostly concentrated in the (normalised) range 0.15–0.35, with a rare tail extending to 0.5–0.6 at the tip. This means that “most boxes are medium-sized and approximately square/oval”.

**Fig. 5:**
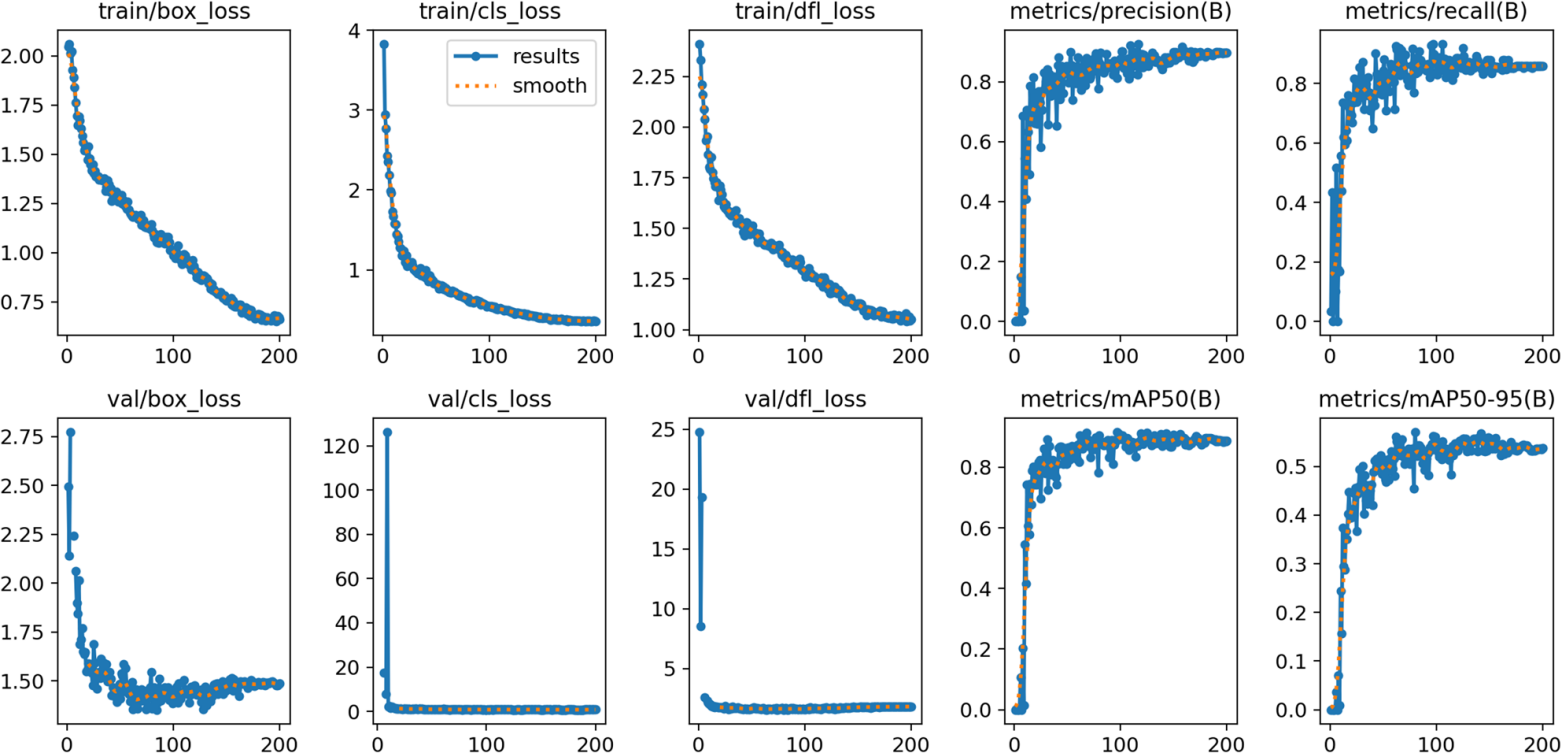
Training/Validation curves of YOLO based lymph node detector

The set of curves shown in Fig. 5 shows that the model converges consistently and significantly over 200 epochs. On the training side, the box_loss (~ 2.0→0.7), cls_loss (~ 3.8→0.5) and DFL (Distribution Focal Loss, ~ 2.3→1.0) components gradually plateau after a rapid initial decrease, indicating that the optimisation is progressing well and the learning rate timing is set appropriately. In particular, the steady decrease in DFL indicates that the centre and size distributions in the box regression are modelled increasingly accurately.

The validation curves present a similar picture. val/box_loss decreases from about 2.7 to 1.4–1.5 and oscillates in a narrow range, while val/cls_loss decreases from an initial head-initialisation-induced peak to < 1 very quickly and then to values close to zero. The fact that val/DFL_loss also decreases sharply and does not show a significant increase in the late epochs indicates that the training-validation gap remains small and there is no obvious sign of overfitting in the late epochs. In other words, the model transfers well what it has learnt in the training set to the validation set in terms of both classification and localisation. Detection metrics support this picture. While the Precision curve reaches ≥ 0.90 and plateaus in a short time, the Recall curve approaches saturation in the 0.85–0.88 band. This combination indicates that the model effectively suppresses false positives (high precision) while missing relatively few targets (high sensitivity). In practical use, this behaviour indicates that a balanced F1 will be obtained by choosing a default confidence threshold (conf) around 0.5–0.6. The summary metrics, mAP@0.5 and mAP@0.5–0.95 curves, show that the model performs at a high level in both class separation and box localisation. mAP@0.5 reaches ~ 0.90 + in the early period and remains stable, indicating that class and coarse localisation are reliable; mAP@0.5–0.95 plateaus at ~ 0.55–0.60, indicating that box precision has room for improvement at very stringent IoU thresholds (0.75–0.95).

**Fig. 6:**
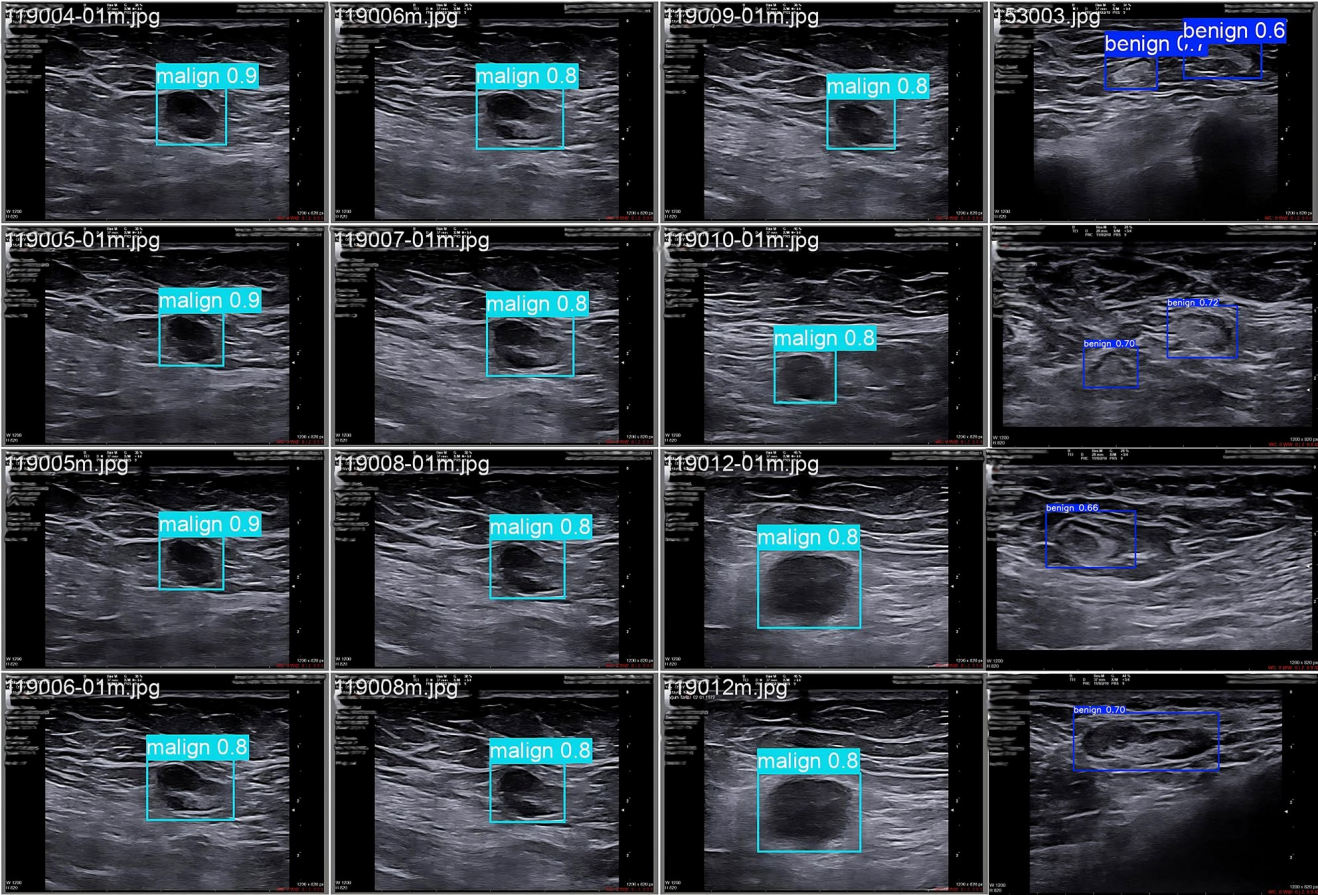
Sample labelling for node detection and classification in the model

Figure 6 shows qualitatively the stability of the model in different field conditions. Image quality, texture pattern and lesion size vary significantly. Nevertheless, the class predictions are mostly concentrated around 0.8. This concentration indicates a sufficient discrimination signal. Box placements cover the central density area of the lesions. Some context from the surrounding tissue is also intentionally left. This preference is practical in single-stage YOLO architectures. This is because class distinction is often reinforced by environmental cues such as stromal striation and fascia transitions. Some specimens show multiple boxes within a single frame. This is especially evident in benign cases. It suggests that the network can separate weak echo structures and iso/hypoechoic foci as separate candidates. It also implies that the NMS (Non-Max Suppression) threshold remains relatively permissive in overlapping benign candidates. In malignant specimens, scores are more stable in the range 0.80–0.90. Boxes are more compact and fit better at the boundaries. This pattern indicates consistent capture of morphological cues such as border irregularity and heterogeneous hypoechoic nuclei.

**Fig. 7:**
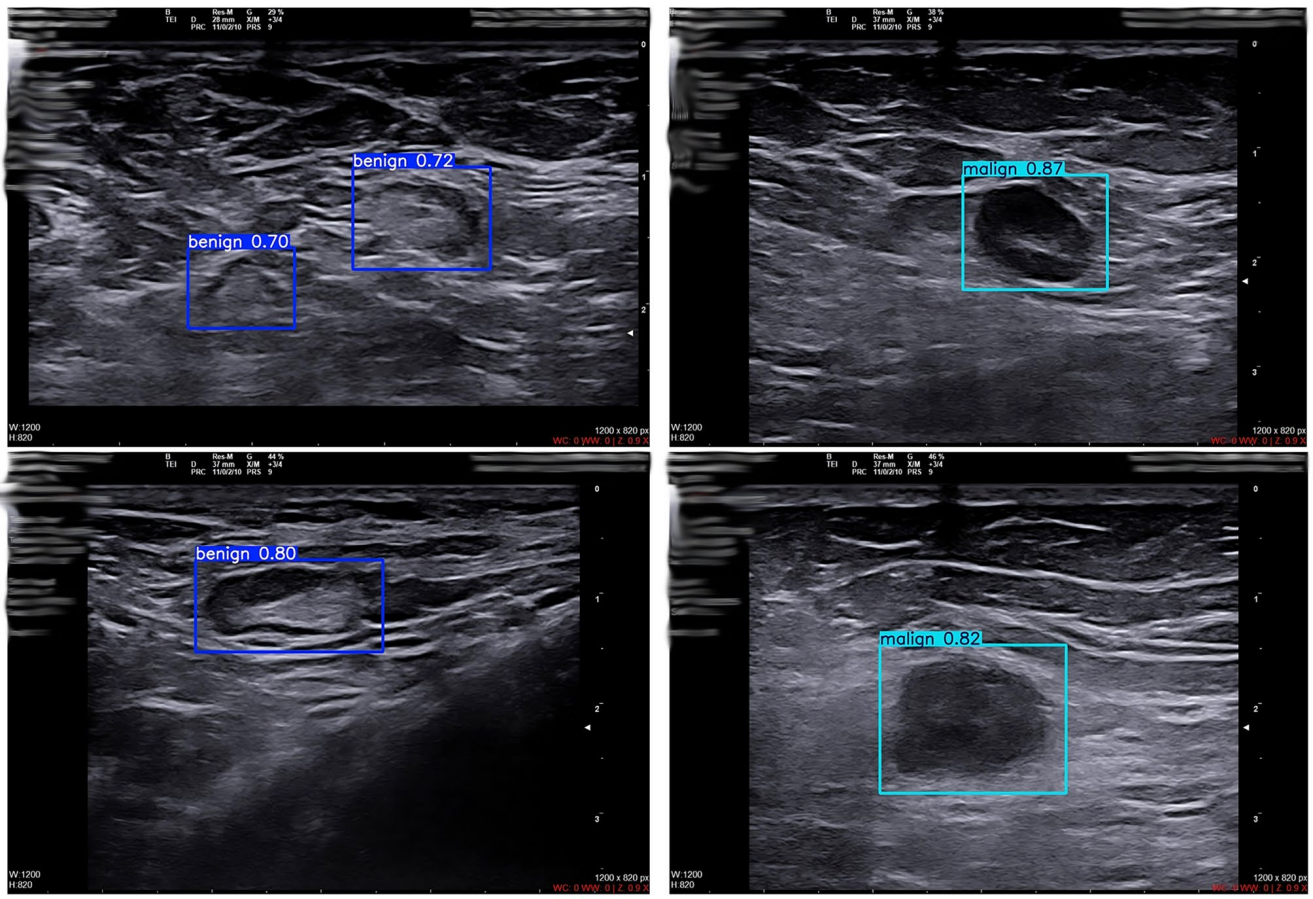
High-performance single and multi-labelling examples

Figure 7 shows the class-specific clues of the model on higher resolution samples. In benign specimens, the boxes follow the long axis of the lesion horizontally. The contours are smooth and ovality is preserved. The box also encompasses the surrounding relatively homogenous echo texture. Confidence scores are in the range 0.70–0.80. This distribution is consistent with the calibration of the PR curves. Helps to limit false positives at a cautious operating threshold.

In malignant specimens, boxes tightly surround foci with irregular contours. Hypoechoic nucleus is more prominent. Internal structure is heterogeneous. Confidence scores are in the range of 0.82–0.87. The model shows high sensitivity to these morphological cues. Box-field overlap is qualitatively high. Small overflows near the short edge are acceptable. These overflows preserve useful context, such as acoustic shadowing and stromal striation. The background speckle pattern is a challenge in both classes. Areas adjacent to the fascia border are also a critical source of error. The expected hierarchy between label and confidence score is maintained in the panel. Irregular and heterogeneous lesions receive higher scores. Uniformly circumscribed and homogeneous lesions are scored more cautiously. This consistency is consistent with the F1-confidence and PR curves. A threshold in the range of 0.5–0.6 provides a balanced sensitivity-sensitivity trade-off for clinical use.

From a clinical perspective, these unmatched detections may correspond to anatomical structures with node-like appearance. Qualitative inspection suggests that vascular cross-sections, fascial planes, or pleural interfaces may occasionally mimic lymph node morphology, leading to false-positive detections.

## Discussion

Today, axillary lymph node dissection and sentinel lymph node biopsy (SLNB) are accepted as standard surgical methods for axillary lymph node staging [18]. However, both operations carry varying degrees of risk for postoperative complications and morbidity. Recent studies have focused on minimising unnecessary axillary surgical interventions and avoiding overtreatment in breast cancer [18]. The Sentinel Node and Observation after Axillary Ultrasound (SOUND) trial demonstrated that in patients with tumor size ≤ 2 cm and negative findings on axillary ultrasonography, omission of SLNB is safe, provided that axillary staging is performed preoperatively with USG [19].

USG is the most commonly preferred imaging method in ALN assessment. However, the diagnostic accuracy of ultrasound largely depends on the radiologist’s experience [20]. Therefore, artificial intelligence-based technological approaches are needed to detect ALN metastasis in a non-invasive, automated and repeatable manner.

Among artificial intelligence methods, deep learning-based models are used in various tasks such as image classification, object detection, segmentation, and image synthesis. Object detection is the process of simultaneously determining both the location and category of objects within an image [21]. With the development of CNNs, models that operate faster and with higher accuracy have been developed in this field. To this end, high-precision object detection architectures such as R-CNN, Spatial Pyramid Pooling (SPP)-Net, Fast R-CNN, Faster R-CNN, You Only Look Once (YOLO), Single Shot MultiBox Detector (SSD), Feature Pyramid Networks (FPN), and RetinaNet have been designed [22].

The effectiveness of deep learning and hybrid systems in medical image analysis has been demonstrated in numerous different pathologies in the literature. For example, high accuracy rates of 99.3% have been achieved in the early diagnosis of oral squamous cell carcinoma (OSCC) using CNN fusion features and hybrid systems [23]. Similarly, combining deep learning features with handcrafted features in the classification of breast cancer histopathological images has significantly improved diagnostic success and achieved a high accuracy of 99.7% at a magnification factor of 400x [24]. High performance values were obtained in the differential diagnosis of malignant lymphomas using feature fusion of models such as MobileNet and VGG16 with the XGBoost algorithm [25]; while in cervical cancer screening, 99% success was achieved using ACA algorithm-based hybrid methods [26]. Furthermore, the integration of radiomic features in predicting diabetic retinopathy stages from fundus images [27] and the use of CNN-ANN hybrid models in analyzing dermoscopic images of skin lesions demonstrate the broad application spectrum of artificial intelligence [28]. Another comprehensive study on breast cancer diagnosis reported a 100% accuracy rate using hybrid and deep learning methods [29].

In this study, a deep learning model based on YOLO-v11 was trained to simultaneously perform lesion localisation and benign-malignant classification on ALN ultrasonography in patients diagnosed with breast cancer. The model’s accuracy rate was determined to be 0.89 for benign lymph nodes and 0.84 for malignant lymph nodes. The mAP@0.5 value calculated under the Precision–Recall (PR) curve was 0.904, with 0.866 in the benign class and 0.942 in the malignant class. The relative deviation observed in malignant cases is thought to stem from the irregular contours of metastatic lymph nodes and the more pronounced hypoechoic cortical thickening. The confidence scores generated by the model ranged from 0.82 to 0.87 and showed consistency with the F1–confidence and PR curves. From a clinical application perspective, the threshold value in the 0.5–0.6 range provides a balanced trade-off between sensitivity and specificity.

According to the literature review and research conducted, there is no study in the literature focusing on the benign–malignant distinction of ALN metastasis using the YOLO-v11 model. In this respect, our study is the first in this field and presents findings that will support the appropriate and practical use of deep learning models in ALN assessment in the future.

In the literature, there are various studies predicting ALN metastasis based on imaging findings of breast masses. The studies by Qiang Guo, Li-Qiang. Z., Liu and colleagues have demonstrated that ultrasonographic features of breast tumours show a strong correlation with ALN metastasis prediction and may contribute to more accurate preoperative prediction of the status of clinically negative lymph nodes [13, 30, 31]. However, the number of studies evaluating ALN directly from lymph node images is quite limited.

Wang, et al. reported an accuracy rate of 88.89%, an AUROC value of 79.99% and an NPV rate of 86.96% in their study [32]. Coronado-Gutiérrez, et al. achieved 86.4% accuracy, 84.9% sensitivity, and 87.7% specificity using 118 lymph node ultrasound images [33]. Ozaki, et al. achieved 94% sensitivity, 88% specificity, and AUC = 0.966 using the Xception architecture. Compared to these findings in the literature, the accuracy (benign 0.89, malignant 0.84) and sensitivity (benign 0.866, malignant 0.942) values obtained in our study are similar and show clinically meaningful performance [34]. On the other hand, Wang, et al. used the YOLO-v7 model in the evaluation of cervical lymph nodes and reported mAP = 96.4% at a 50% IoU threshold, accuracy values of 0.962 and 0.960 for benign and metastatic lymph nodes, respectively, and precision values of 0.928 and 0.927 [35]. Although the accuracy and precision values obtained in our study are relatively lower than those in this study, they demonstrate acceptable performance when considering differences in data diversity, image quality, and disease region.

In radiomic analysis methods, the segmentation process is typically based on manual drawing. This method is time-consuming and prone to human error [36]. In contrast, the model proposed in this study offers a more practical solution that does not require inference and can perform automatic prediction and labelling. The model’s accuracy, precision, sensitivity, and F1 score values support the system’s usability in clinical practice.

One of the main challenges observed during evaluation was the presence of unmatched detections arising from background anatomical structures. USG images obtained in clinical settings often contain patient information, scale bars, and lesion markers. Therefore, these elements were deliberately preserved during the training of the YOLO-v11 model. Although this approach causes some noise during the training phase, it is necessary for developing a model that is applicable in real clinical conditions.

An important observation in our results is the higher mAP@0.5 performance for malignant lymph nodes (0.942) compared to benign nodes (0.866). This asymmetry was not introduced intentionally through class weighting or loss manipulation. Instead, the model was trained using balanced class distributions without differential penalty assignment. The higher performance in malignant cases likely reflects the more pronounced morphological characteristics of metastatic lymph nodes, such as cortical thickening, irregular margins, and heterogeneous hypoechoic structure, which provide stronger discriminative cues for deep feature extraction. From a clinical perspective, this asymmetry is aligned with diagnostic priorities. Missing a malignant lymph node (false negative) has significantly greater clinical consequences than overcalling a benign node (false positive). Therefore, the model’s higher sensitivity and detection confidence for malignant cases may be considered clinically advantageous. Moreover, threshold analysis demonstrated that sensitivity for malignant detection remains high within the recommended confidence band (0.52–0.60), allowing clinicians to prioritize malignancy detection while maintaining acceptable specificity. Future studies may explore cost-sensitive learning or class-weighted loss functions to explicitly optimize malignancy sensitivity in accordance with clinical risk tolerance.

Our study has several limitations. First, this was a single-centre study using data acquired from two ultrasound systems within the same institution. Although internal validation was performed, image characteristics, acquisition protocols, and patient demographics may differ across institutions. Therefore, the current findings primarily reflect internal performance rather than full real-world generalizability. Before clinical deployment, multicentre external validation on independent datasets from different institutions, operators, and ultrasound manufacturers is essential to confirm robustness and reproducibility. Future work will focus on prospective, multicentre validation studies and cross-device domain adaptation strategies to ensure safe clinical translation.

Another limitation of this study is the relatively small independent test set. Although patient-level splitting was strictly applied to prevent data leakage, the limited number of test images may restrict statistical generalizability. Confidence intervals were therefore calculated using bootstrap resampling to quantify metric uncertainty. Furthermore, no external validation dataset from an independent institution was available. Future multicentre studies with external validation cohorts are necessary to confirm model robustness across different devices and clinical environments.

## Conclusion

In this study, the YOLO-v11 model demonstrated high and clinically meaningful performance in detecting and classifying axillary lymph nodes in ultrasound images. Although a direct comparative evaluation with other deep learning architectures and radiologist performance on the same dataset was not performed, the results obtained are similar in nature to those reported in previous studies in the literature. The model’s performance has been validated using standard metrics, and the results obtained demonstrate the model’s applicability in clinical practice. In this regard, the study offers a fast, reliable, and non-invasive technological approach that can provide decision support to radiologists and clinicians.

### Ethical approval and data privacy

This study was conducted with the approval of the Scientific Research and Publication Ethics Board of İzmir Katip Çelebi University of Health Research Ethics Committee, under the official correspondence dated 09/10/2025 (Decision No: 0596). Written informed consent was obtained from all participants, who voluntarily agreed to participate in the study and to allow the use of their data for scientific purposes. All collected data were anonymized, and no personal identifiable information was used during the analysis. Data processing was performed solely using hidden patient identifiers. The research was carried out in full compliance with the principles of the Declaration of Helsinki.

## Data Availability

Reasonable requests may be made with the author’s permission.

## Abbreviations

ALN: Axillary lymph node
USG: Ultrasound
PR: Precision Recall
IARC: International Agency for Research on Cancer
ACOSOG: American College of Surgeons Oncology Group
SLNB: Sentinel Lymph Node biopsy
CNN: Convolutional Neural Network

## References

[1] Sung H, Ferlay J, Siegel RL, et al. Global cancer statistics 2020: GLOBOCAN estimates of incidence and mortality worldwide for 36 cancers in 185 countries. CA Cancer J Clin. 2021;71(3):209–49. 10.3322/caac.21660. 10.3322/caac.21660 33538338

[2] Miao H, Hartman M, Bhoo-Pathy N, et al. Predicting survival of de novo metastatic breast cancer in Asian women: systematic review and validation study. PLoS ONE. 2014;3(4):e93755. 10.1371/journal.pone.0093755. 10.1371/journal.pone.0093755 PMC3973579 24695692

[3] Wu T, Long Q, Zeng L, et al. Axillary lymph node metastasis in breast cancer: from historical axillary surgery to preoperative diagnosis and current advances in axillary management. BMC Surg. 2025;25:81. 10.1186/s12893-025-02802-2. 40016717 10.1186/s12893-025-02802-2 PMC11869450

[4] Choi HG, Park M, Seo M, et al. Preoperative Axillary Lymph Node Evaluation in Breast Cancer: Current Issues and Literature Review. Ultrasonography Q Febr. 2017;33(1):1–8. 10.1097/RUQ.0000000000000277. 10.1097/RUQ.0000000000000277 28187012

[5] Valente SA, Levine GM, Silverstein MJ, et al. Accuracy of predicting axillary lymph node positivity by physical examination, mammography, ultrasonography, and magnetic resonance imaging. Ann Surg Oncol. 2012;19(6):1825–30. 10.1245/s10434-011-2200-7. 22227922 10.1245/s10434-011-2200-7

[6] Chatterjee A, Serniak N, Czerniecki BJ. Sentinel Lymph Node Biopsy in Breast Cancer: A Work in Progress. Cancer J. 2015;21(1):7–10. 10.1097/PPO.0000000000000090. 25611773 10.1097/PPO.0000000000000090 PMC4304410

[7] Giuliano AE, Ballman KV, McCall L, et al. Morrow M.Effect of Axillary Dissection vs No Axillary Dissection on 10-Year Overall Survival Among Women With Invasive Breast Cancer and Sentinel Node Metastasis: The ACOSOG Z0011 (Alliance) Randomized Clinical Trial. JAMA. 2017;12(10):918–26. 10.1001/jama.2017.11470. 10.1001/jama.2017.11470 PMC5672806 28898379

[8] Gradishar WJ, Moran MS, Abraham J, et al. Breast Cancer, Version 3.2024, NCCN Clinical Practice Guidelines in Oncology. J Natl Compr Canc Netw. 2024;22(5):331–57. 10.6004/jnccn.2024.0035. 39019058 10.6004/jnccn.2024.0035

[9] Zheng H, Zhao R, Wang W, et al. The accuracy of ultrasound-guided fine-needle aspiration and core needle biopsy in diagnosing axillary lymph nodes in women with breast cancer: a systematic review and meta-analysis. Front Oncol. 2023;13:1166035. 10.3389/fonc.2023.1166035. 37416528 10.3389/fonc.2023.1166035 PMC10320388

[10] Pinheiro PdaC, Elias DJ, Pinto Nazário S. Axillary lymph nodes in breast cancer patients: sonographic evaluation. Radiologia Brasileira. 2014;47(4):240–4. 10.1590/0100-3984.2013.1689. 25741091 10.1590/0100-3984.2013.1689 PMC4337126

[11] Zhang YN, Wang CJ, Xu Y, et al. Sensitivity, specificity, and accuracy of ultrasound in diagnosis of breast cancer metastasis to axillary lymph nodes in Chinese patients. Ultrasound Med Biol. 2015;41(7):1835–41. 10.1016/j.ultrasmedbio.2015.03.024. 25933712 10.1016/j.ultrasmedbio.2015.03.024

[12] Lu G, Tian R, Yang W, et al. Deep learning radiomics based on multimodal imaging for distinguishing benign and malignant breast tumours. Front Med (Lausanne). 2024;5:11:1402967. 10.3389/fmed.2024.1402967. 10.3389/fmed.2024.1402967 PMC11257849 39036101

[13] Lu H, Zou L, Xu N, et al. Deep learning radiomics based prediction of axillary lymph node metastasis in breast cancer. NPJ Breast Cancer. 2024;10(1):54. 10.1038/s41523-024-00628-4. 38472210 10.1038/s41523-024-00628-4 PMC10933422

[14] Liu W, Li L, Deng J, et al. Ultrasound-based radiomics model for predicting axillary lymph node metastasis of breast cancer. BMC Med Imaging. 2025;25:434. 10.1186/s12880-025-01978-6. 41162897 10.1186/s12880-025-01978-6 PMC12573990

[15] Sun Yibo S, Zhe C. The evolution of object detection methods. Eng Appl Artif Intell. 2024;133:108458. 10.1016/j.engappai.2024.108458.

[16] Sharma A, Jain A, Sinha A. Comparative analysis of deep learning image detection algorithms. J Big Data. 2021;8(1):66. 10.1186/s40537-021-00434-w.

[17] Muhammed AD, Ekmekçi D. Breast cancer diagnosis using YOLO-based multiscale parallel CNN and flattened threshold Swish. Appl Sci (Basel). 2024;22(7):2680. 10.3390/app14072680.

[18] Fancellu A, Cottu P, Gentilini O, et al. De-escalation of axillary treatment in early breast cancer — A narrative review. Transl Breast Cancer Res. 2025;1:6. 10.21037/tbcr-24-45. 10.21037/tbcr-24-45 PMC11836741 39980812

[19] Gentilini O, Veronesi U, Peccatori FA, et al. Sentinel Lymph Node Biopsy vs No Axillary Surgery in Patients With Small Breast Cancer and Negative Results on Ultrasonography of Axillary Lymph Nodes (SOUND): A Randomized Clinical Trial. JAMA Oncol. 2023;9(11):1622–31. 10.1001/jamaoncol.2023.3759 PMC10514873 37733364

[20] Rezvani A, Zahergivar A, Iranpour P, et al. Diagnostic accuracy of axillary ultrasonography compared with intraoperative pathological findings in patients with breast cancer. Asian Pac J Cancer Prev. 2018;19(12):3615–21. 10.31557/APJCP.2018.19.12.3615. 30583690 10.31557/APJCP.2018.19.12.3615 PMC6428527

[21] Carriero A, Groenhoff L, Vologina E, et al. Deep learning in breast cancer imaging: state of the art and recent advances as of 2024. Diagnostics (Basel). 2024;14(8):848. 10.3390/diagnostics14080848. 38667493 10.3390/diagnostics14080848 PMC11048882

[22] Yang R, Yu Y. Artificial Convolutional Neural Network in Object Detection and Semantic Segmentation for Medical Imaging Analysis. Front Oncol. 2021;9:11:638182. 10.3389/fonc.2021.638182. 10.3389/fonc.2021.638182 PMC7986719 33768000

[23] Ahmed IA, Senan EM, Shatnawi HSA. Analysis of histopathological images for early diagnosis of oral squamous cell carcinoma by hybrid systems based on CNN fusion features. Int J Intell Syst. 2023.

[24] Al-Jabbar M, Alshahrani M, Senan EM, Ahmed IA. Analyzing histological images using hybrid techniques for early detection of multi-class breast cancer based on fusion features of CNN and handcrafted. Diagnostics. 2023. 10.3390/diagnostics13101753 PMC10217631 37238243

[25] Hamdi M, Senan EM, Jadhav ME, Olayah F, Awaji B, Alalayah KM. Hybrid models based on fusion features of a CNN and handcrafted features for accurate histopathological image analysis for diagnosing malignant lymphomas. Diagnostics. 2023. 10.3390/diagnostics13132258 PMC10341222 37443652

[26] Hamdi M, Senan EM, Awaji B, Olayah F, Jadhav ME, Alalayah KM. Analysis of WSI images by hybrid systems with fusion features for early diagnosis of cervical cancer. Diagnostics. 2023. 10.3390/diagnostics13152538 PMC10416962 37568901

[27] Shamsan A, Senan EM, Ahmad Shatnawi HS. Predicting of diabetic retinopathy development stages of fundus images using deep learning based on combined features. PLoS ONE. 2023. 10.1371/journal.pone.0289555 PMC10588832 37862328

[28] Ahmed IA, Senan EM, Shatnawi HSA, Alkhraisha ZM, Al-Azzam MMA. Multi-models of analyzing dermoscopy images for early detection of multi-class skin lesions based on fused features. Processes. 2023.

[29] Al-Jabbar M, Alshahrani M, Senan EM, Ahmed IA. Multi-method diagnosis of histopathological images for early detection of breast cancer based on hybrid and deep learning. Mathematics. 2023.

[30] Guo Q, Zhang LQ, Liu M, et al. Ultrasound Features of Breast Cancer for Predicting Axillary Lymph Node Metastasis. J Ultrasound Med. 2018;37(6):1354–63. 10.1002/jum.14469. 29119589 10.1002/jum.14469

[31] Zhou LQ, Wu XL, Huang SY, et al. Lymph node metastasis prediction from primary breast cancer US images using deep learning. Radiology. 2020;294(1):19–28. 10.1148/radiol.2019190372. 31746687 10.1148/radiol.2019190372

[32] Wang X, Nie L, Zhu Q, et al. Artificial intelligence-assisted ultrasound for the non-invasive prediction of axillary lymph node metastasis in breast cancer. BMC Cancer. 2024;24:910. 10.1186/s12885-024-12619-6. 39075447 10.1186/s12885-024-12619-6 PMC11285453

[33] Coronado-Gutiérrez D, Santamaría G, Ganau S, et al. Quantitative ultrasound image analysis of axillary lymph nodes to diagnose metastatic involvement in breast cancer. Ultrasound Med Biol. 2019;45(11):2932–41. 31444031 10.1016/j.ultrasmedbio.2019.07.413

[34] Ozaki J, Fujioka T, Yamaga E, Hayashi A, Kujiraoka Y, Imokawa T, Takahashi K, Okawa S, Yashima Y, Mori M, Kubota K, Oda G, Nakagawa T, Tateishi U. Deep learning method with a convolutional neural network for image classification of normal and metastatic axillary lymph nodes on breast ultrasonography. Jpn J Radiol. 2022;40(8):814–22. 10.1007/s11604-022-01261-6. 35284996 10.1007/s11604-022-01261-6

[35] Wang Y, Yang C, Yang Q, et al. Diagnosis of cervical lymphoma using a YOLO-v7-based model with transfer learning. Sci Rep. 2024;14:11073. 10.1038/s41598-024-61955-x. 38744888 10.1038/s41598-024-61955-x PMC11094110

[36] Van Timmeren JE, Cester D, Tanadini-Lang S, et al. Radiomics in medical imaging—How-to guide and critical reflection. Insights Imaging. 2020;11(1):91. 10.1186/s13244-020-00887-2. 32785796 10.1186/s13244-020-00887-2 PMC7423816

